# Ni(II)-Salophen—Comprehensive Analysis on Electrochemical and Spectral Characterization and Biological Studies

**DOI:** 10.3390/molecules28145464

**Published:** 2023-07-17

**Authors:** Francis Aurelien Ngounoue Kamga, Madalina-Marina Hrubaru, Oana Enache, Elena Diacu, Constantin Draghici, Victorita Tecuceanu, Eleonora-Mihaela Ungureanu, Stephanie Nkemone, Peter T. Ndifon

**Affiliations:** 1 Coordination Chemistry Laboratory, Department of Inorganic Chemistry, Faculty of Science, University of Yaounde 1, Yaounde P.O. Box 812, Cameroon; fr.kamga@gmail.com (F.A.N.K.); cristina.nicolescu.profa@gmail.com (S.N.); pndifon@facsciences-uy1.cm (P.T.N.); 2 Faculty of Chemical Engineering and Biotechnologies, University “Politehnica” of Bucharest, Gheorghe Polizu 1–7, Sector 1, 011061 Bucharest, Romania; madalina_marina@yahoo.com (M.-M.H.); oana.enache18@yahoo.com (O.E.); elena_diacu@yahoo.co.uk (E.D.); 3 “C. D. Nenitzescu” Institute of Organic and Supramolecular Chemistry, Romanian Academy, Sector 6, Spl. Independentei 202B, 060023 Bucharest, Romania; cst_drag@yahoo.com (C.D.); vichi_tecu@yahoo.com (V.T.)

**Keywords:** Ni(II)-salophen, electrochemical characterization, chemically modified electrodes based on Ni(II)-salophen, antimicrobial activity, antioxidant activity

## Abstract

New aspects of the Ni(II)-salophen complex and salophen ligand precursor were found during deep electrochemical and optical characterization, as well as biological studies for new pharmacological applications. Physicochemical and spectroscopic methods (^1^H- and ^13^C-NMR, FT-IR and UV-Vis, electrospray ionization mass spectroscopy, thermogravimetric analysis, and molar conductance measurements) were also used to prove that the salophen ligand acts as a tetradentate and coordinates to the central metal through nitrogen and oxygen atoms. The electrochemical behavior of the free Schiff salophen ligand (H_2_**L**) and its Ni(II) complex (Ni(II)**L**) was deeply studied in tetrabutylammonium perchlorate solutions in acetonitrile via CV, DPV, and RDE. Blue films on the surfaces of the electrodes as a result of the electropolymerization processes were put in evidence and characterized via CV and DPV. (H_2_**L**) and Ni(II)**L** complexes were tested for their antimicrobial, antifungal, and antioxidant activity, showing good antimicrobial and antifungal activity against several bacteria and fungi.

## 1. Introduction

**Figure 1:**
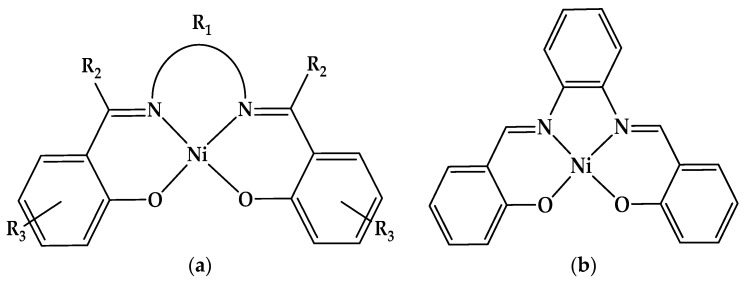
(**a**) Structure of Ni(II)-salen complexes, where R_1_ is a two–four carbon-atom-saturated bridge or a substituted aromatic moiety, R_2_ is H or an alkyl substituent, and R_3_ is a halogen or another substituent to the aromatic ring; (**b**) Ni(II)-salophen complex studied in the present paper (R_1_ = C_6_H_4_, R_2_ = H, and R_3_ = H).

Over the past few decades, transition metal complexes containing N_2_O_2_ coordination sites, such as salicylidenes [1], have aroused the increased interest of researchers due to their multiple applications in topical fields such as non-linear optics [2], molecular magnetism [3], functional materials and organic electronics [4], light-emitting diodes (OLEDs) [1,5], and display magnetic properties [6]. Among them, salen-like ligands (Figure 1a) were studied for their properties and applications in biology [7], catalysis [8], and electrocatalysis [9]. They are diimines prepared from differently substituted aldehydes and various 1,2 diamines. *N*,*N*’-Bis-(2-hydroxybenzylidene)-benzene-1,2-diamine (H_2_**L**) and its Ni(II) complex (Ni(II)**L** (Figure 1a) were studied using many techniques and in various conditions. Connections with previous results [10] were made. Studying the relationship between the chemical structures and characteristics of Schiff bases and their complexes is very important for understanding their various forms of activity [11]. Numerous studies have focused on the electrochemical behavior of salen ligands and their metal complexes on the relation between electrochemical potential and structure [12,13]. The electrochemical behavior of Schiff bases has been investigated particularly in dipolar aprotic solvents, where the hydrolysis of the iminic moiety is impeded [14,15]. This involves the hydrogenation of the >C=N bond, with the formation of saturated amines or dimeric diamines in several cases [16]. The overall two-electron process gives rise to a single reduction wave or it takes place in two separate one-electron steps, depending on the structure of the substrate and on the proton-donor ability of the medium [15,17].

On the other hand, nickel complexes are powerful catalysts in the chemical and electrochemical reduction of alkyl halides [18,19,20,21]. It has been established that the essential factor in all catalytic processes is the reduction of the transition metal to a low oxidation state and that the catalytic efficiency depends on the capacity of the reducibility of the metal center as well as on the reactivity of the reduced metal complex. In particular, numerous Ni(II)-**salen** complexes have been evaluated via voltammetry electropolymerization [22]. Several of these studies have essentially focused on the electrocatalytic reduction activity of Schiff base complexes in a large number of homogeneous and heterogeneous reactions [23,24,25]. This study invites the further exploration of **L** and its nickel complex Ni(II)**L** (Figure 1b), as they have enormous potential for broader applications that have not been explored in their current uses.

Precisely from this perspective, this paper focuses on the characterization of **L** and its tetradentate Ni(II)**L** complex using different physicochemical and spectroscopic methods. An in-depth electrochemistry characterization of **L** and Ni(II)**L** was carried out to study the electropolymerization mechanism and characteristics of the polymer films deposited on the electrode surface, as well as the behavior of the modified electrodes covered by the electroactive polymer films. The biological properties of these compounds were also tested for antibacterial and antifungal activities against several usual pathogenic strains. The radical scavenging method was used to put in evidence the efficiency of their antioxidant activity. Conductivity studies were also performed.

## 2. Results

The ligand H_2_**L** and Ni(II)**L** complexes were synthesized and confirmed via spectroscopic analyses (NMR, IR, MS, UV-Vis), as will be shown in the paragraph about materials. They were deeply investigated from an electrochemical point of view. Conductometric and thermogravimetric analyses and antimicrobial and antioxidant activity studies were performed as well.

### 2.1. Electrochemical Studies of Salophen Ligand L and its Ni(II) Complex

The electrochemical behavior of H_2_**L** and Ni(II)**L** was studied via cyclic voltammetry (CV), differential pulse voltammetry (DPV), and rotating disk electrode voltammetry (RDE) in acetonitrile containing 0.1 M tetrabutylammonium perchlorate (TBAP) as the supporting electrolyte on a glassy carbon (GC) electrode in a potential range of −3 V to +3 V vs. the Ag/Ag^+^ reference electrode and starting from the stationary potential. After the experiments, the potential axis referred to the ferrocene/ferrocenium (Fc/Fc^+^) redox couple, and the resulting curves are consequently shown. The peaks are denoted in the order of their appearance in DPV and CV direct scans.

#### 2.1.1. Electrochemical Study of Salophen Ligand L

**Figure 2:**
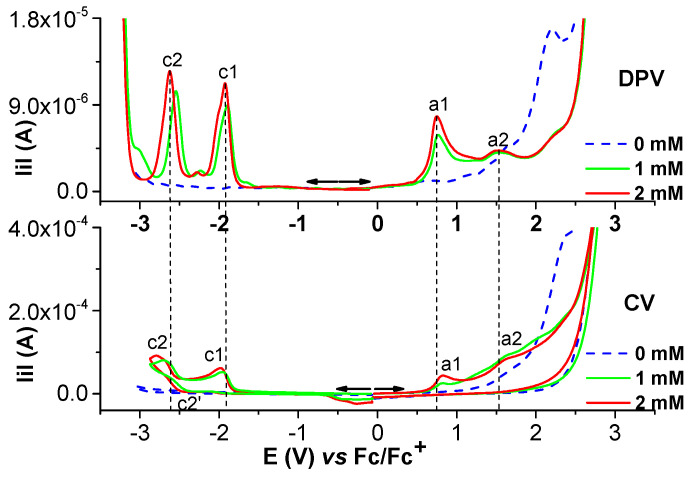
Anodic and cathodic DPV (0.01 V/s) and CV (0.1 V/s) curves on GC of the ligand (H_2_**L**) at different concentrations (mM) in 0.1 M TBAP/CH_3_CN; the cathodic currents are shown in absolute values.

**Figure 3:**
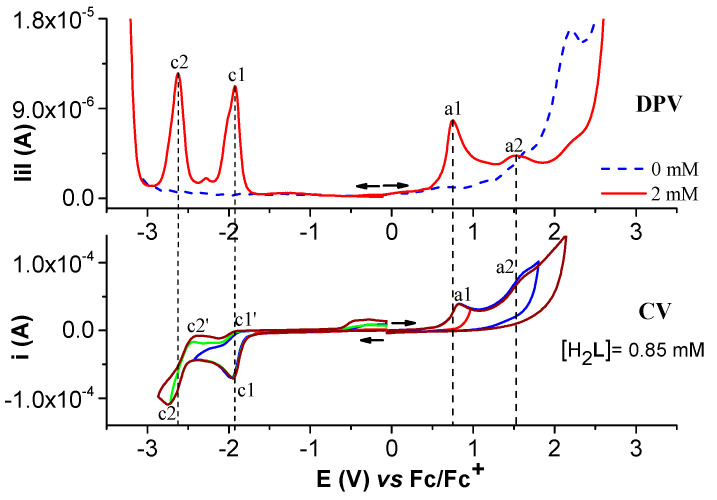
Anodic and cathodic curves from DPV (**up**) and CV (**down**) in different scan domains at 0.1 V/s, and (**down**) experiments in H_2_**L** (2 mM) solution in 0.1 M TBAP/CH_3_CN. Domains cathodic: −2.9 V (brown); −2.7 V (green); −2.5 V (blue). Domains anodic: 1.1 V (red); 1.9 V (blue); 2.4 V (brown).

**Figure 4:**
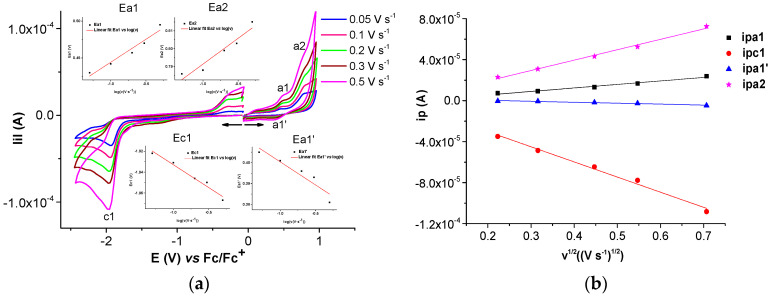
(**a**) Anodic and cathodic cyclic voltammograms at different scan rates for solution of [H_2_**L**] = 0.85 mM in 0.1 M TBAP/CH_3_CN and linear dependences of c1, a1, a1′ and a2 peak potentials on the logarithm of the scan rate (insets Ea1, Ea1′, Ea2 and Ec1, respectively); (**b**) linear dependences of c1, a1, a1′ and a2 peak currents on the square root of the scan rate.

**Figure 5:**
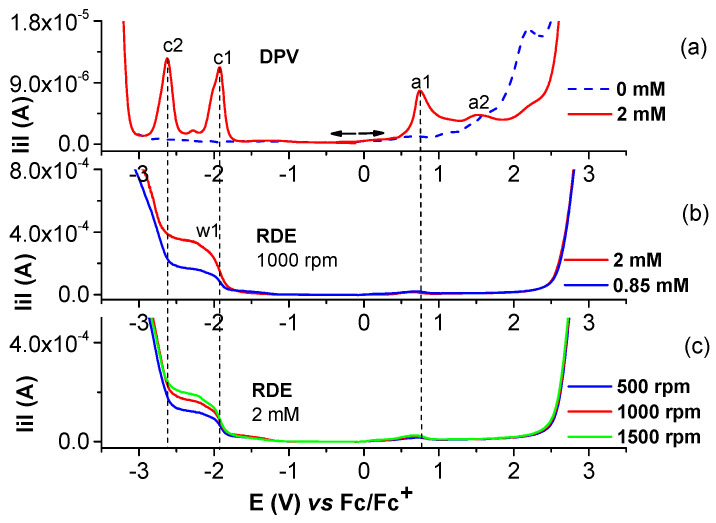
(**a**) DPV curve at 2 mM H_2_**L** in 0.1 M TBAP/CH_3_CN; (**b**) RDE curves at 1000 rpm at different concentrations of H_2_**L** in 0.1 M TBAP/CH_3_CN; (**c**) RDE curves on GC at different rotation rates (rpm) for [H_2_**L**] = 2 mM in 0.1 M TBAP/CH_3_CN; the cathodic currents are shown in absolute values.

Selected anodic and cathodic DPV, CV, and RDE curves recorded in millimolar solutions of (H_2_**L**) are shown in Figure 2, Figure 3, Figure 4 and Figure 5. In the anodic DPV scans, two main peaks were noticed (denoted as a1 and a2 in Figure 2). In the cathodic DPV scans, two peaks were also noticed (denoted as c1 and c2 in Figure 2). The study of CV in different scan domains (Figure 3) and at different scan rates (Figure 4) revealed other peaks: a1′, associated with a1 in the anodic scans, and c1′ and c2′, associated with c1 and c2, respectively, in the cathodic scans. The peak potential and current dependences on the scan rate are shown in the Figure 4 insets. The values of the peak potentials vs. Fc/Fc^+^ from the CV and DPV curves measured for the H_2_**L** solution (1 mM) are given in Tables S1 and S2.

Figure 5 illustrates, in parallel with the DPV curves, the RDE curves obtained at different concentrations of H_2_**L** at a rotation rate of 1000 rpm and the resulting RDE curves for the concentration of 0.85 mM and 2 mM at different rotation rates of the electrode.

#### 2.1.2. Electrochemical Study of Ni(II)L Complex

**Figure 6:**
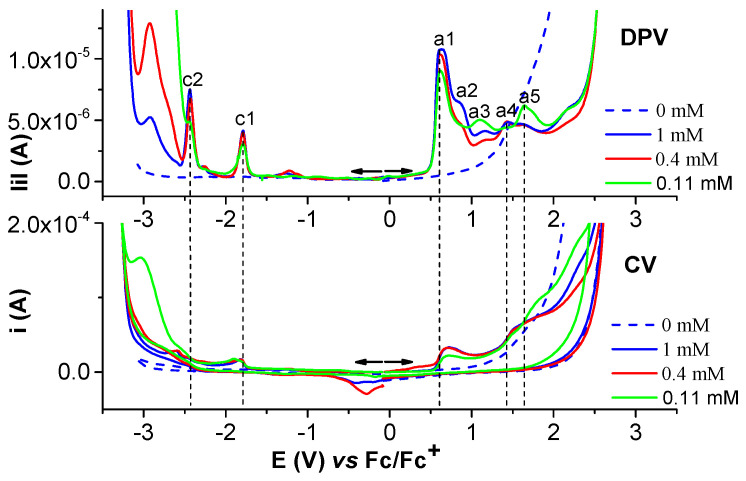
Anodic and cathodic DPV (**up**) and CV at 0.1 V/s (**down**) curves on GC of Ni(II)**L** complex at different concentrations (mM) in 0.1 M TBAP/CH_3_CN; the cathodic currents are shown in absolute values.

**Figure 7:**
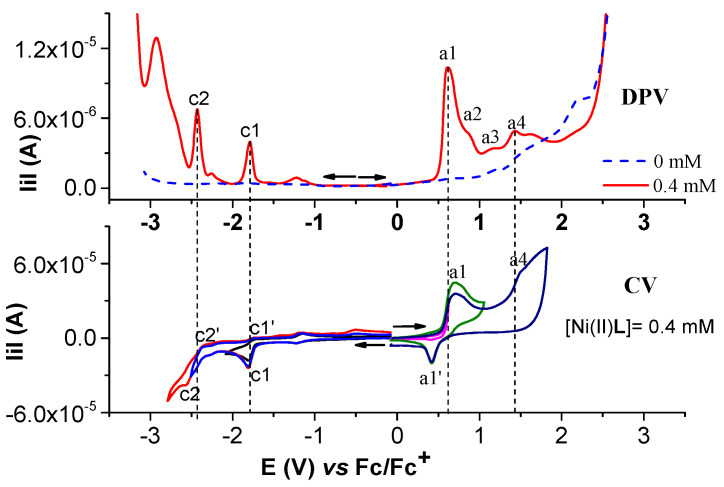
Anodic and cathodic DPV (**up**) and CV in different scan domains at 0.1 V/s (**down**) curves for Ni(II)**L** complex (1 mM) in 0.1 M TBAP/CH_3_CN.

**Figure 8:**
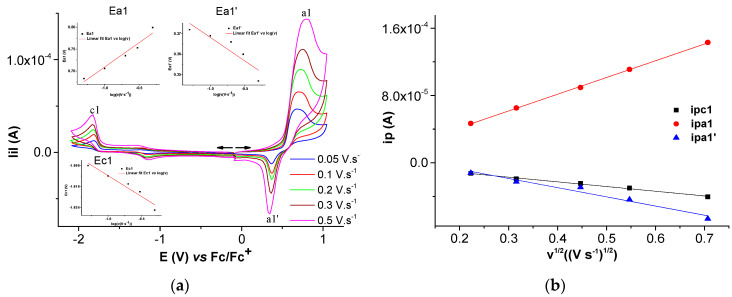
(**a**) Anodic and cathodic cyclic voltammograms at different scan rates for solution of [Ni(II)**L**] = 0.4 mM in 0.1 M TBAP/CH_3_CN and linear dependences of a1, a1′, and c1 peak potentials on the logarithm of the scan rate (insets Ea1, Ea1′, and Ec1, respectively); (**b**) linear dependences of c1, a1, and a1′ peak currents on the square root of the scan rate.

**Figure 9:**
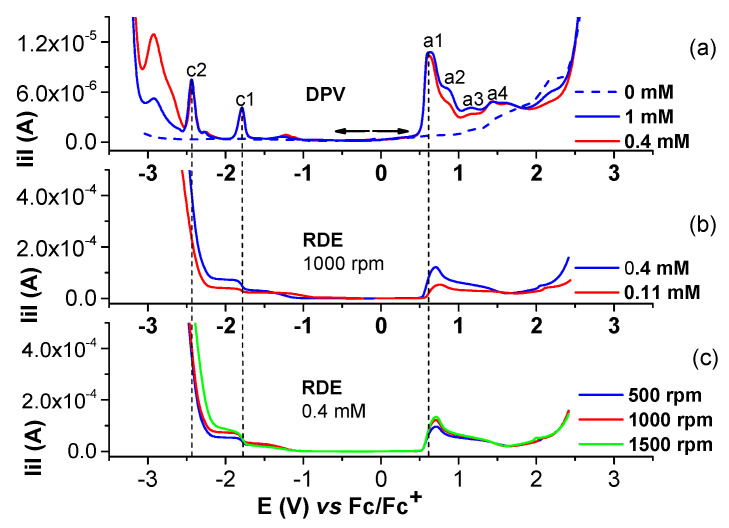
(**a**) DPV curve at 1 mM H_2_**L** in 0.1 M TBAP/CH_3_CN; (**b**) RDE curves at 1000 rpm at different concentrations of Ni(II)**L** in 0.1 M TBAP/CH_3_CN; (**c**) RDE curves on GC at different rotation rates (rpm) for [Ni(II)**L**] = 0.4 mM in 0.1 M TBAP/CH_3_CN; the cathodic currents are shown in absolute values.

Selected anodic and cathodic CV, DPV, and RDE curves recorded in millimolar solutions of Ni(II)**L** are shown in Figure 6, Figure 7, Figure 8 and Figure 9. The values of the peak potential of the CV and DPV curves measured for the Ni(II)**L** solution are given in Tables S3 and S4. The study of CV in different scan domains (Figure 7) and the study at different scan rates (Figure 8a) revealed the peak potential and the current dependences, which are shown in the Figure 8a insets and Figure 8b. The values of the peak potentials vs. Fc/Fc+ from the CV and DPV curves measured for the Ni(II)**L** solution (0.4 mM) are also presented in Table S6. The RDE curves obtained in the study of Ni(II)**L** are shown in parallel with the DPV curves to correlate the processes that were highlighted. Figure 9 illustrates the RDE curves obtained for different solutions of Ni(II)**L** at a rotation rate of 1000 rpm and the resulting RDE curves for the concentration of 0.4 mM and 0.11 mM at different rotation rates of the electrode.

### 2.2. Film Formation from Ni(II)L Complexes and Their Electrochemical Characterization

**Figure 10:**
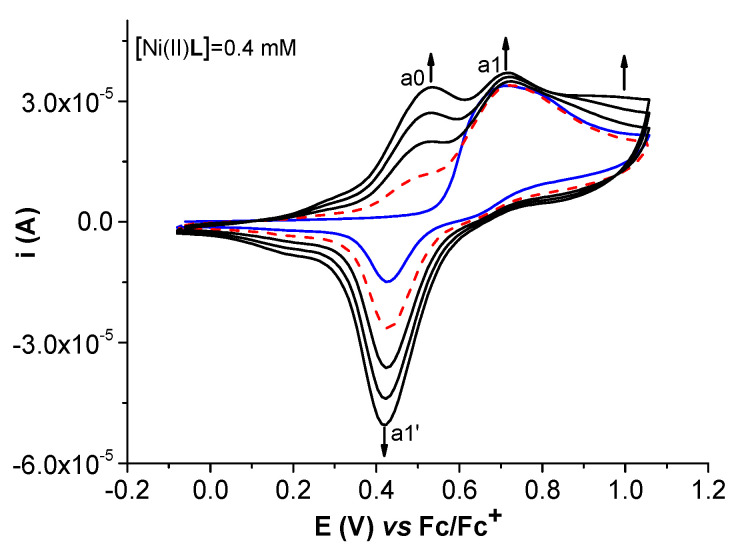
CVs (0.1 V/s) of Ni(II)**L** (0.4 mM) in TBAP/CH_3_CN at 1.13 V: solid blue line—1st cycle of electropolymerization process of Ni(II)**L**; dashed red line—2nd cycle.

**Figure 11:**
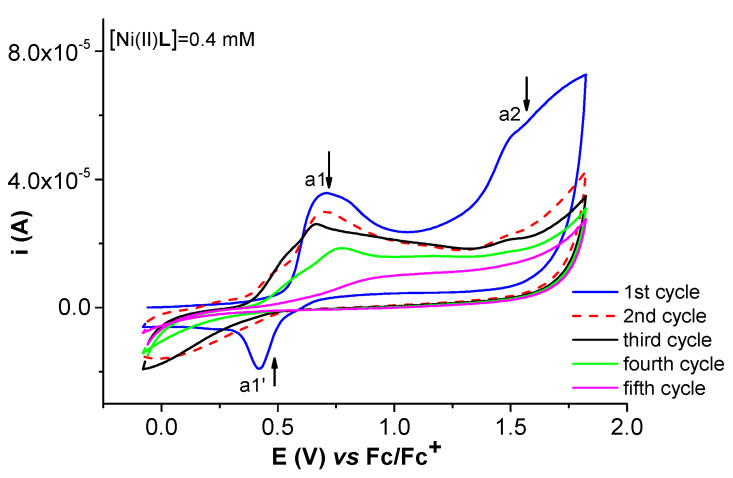
CVs (0.1 V/s) of Ni(II)**L** (0.4 mM) in TBAP/CH_3_CN at 1.19 V: solid blue line—1st cycle of electropolymerization process of Ni(II)**L**; dashed red line—2nd cycle.

**Figure 12:**
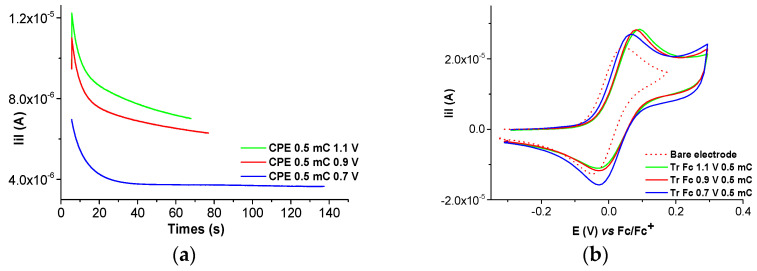
(**a**) Chronoamperograms during the preparation of Ni(II)**L**-CMEs in 0.4 mM solution of Ni(II)**L** in 0.1 M TBAP/CH_3_CN via CPE at different potentials; (**b**) CV curves (0.1 V/s) after their transfer in ferrocene solution (1 mM) in 0.1 M TBAP/CH_3_CN vs. bare electrode.

**Figure 13:**
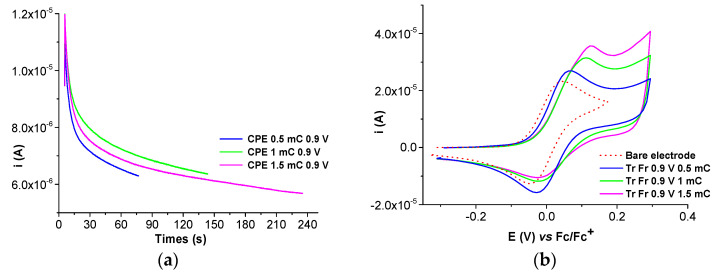
(**a**) Chronoamperograms during the preparation of Ni(II)**L**-CMEs in 0.4 mM solution of Ni(II)**L** in 0.1 M TBAP/CH_3_CN via CPE at different charges; (**b**) CV curves (0.1 V/s) after their transfer in ferrocene solution (1 mM) in 0.1 M TBAP/CH_3_CN vs. bare electrode.

The electrochemical behavior of millimolar solutions of the Ni(II)**L** complex shows that film formation occurred, leading to modified electrodes. This process was studied via CV, DPV, and chronoamperometry in millimolar solutions of Ni(II)**L** in 0.1 M TBAP/CH_3_CN. The modified electrodes obtained were transferred in the supporting electrolyte (0.1 TBAP/CH_3_CN) and in ferrocene solution in the supporting electrolyte, and their CV and DPV curves were recorded and compared to the corresponding curve obtained via the bare electrode. The changes due to the presence of the films on the electrode were examined. The films were obtained either by scanning (Figure 10 and Figure 11) or by controlled potential electrolysis (Figure 12a, Figure 13a and Figure S2a). The chronoamperometry indicates good reproducibility in film preparation via CPE (Figure S2).

**Figure 14:**
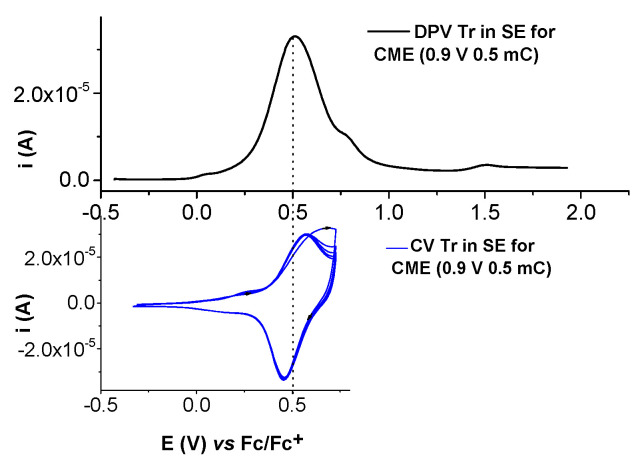
Characterization of Ni(II)**L**-CMEs (prepared via CPE (0.9 V, 0.5 mC) in a solution of Ni(II)**L** (0.11 mM) in 0.1 M TBAP/CH_3_CN) in the supporting electrolyte (0.1 M TBAP/CH_3_CN) via DPV (**up**) and CV (0.1 V/s)—5 successive curves (**down**).

**Figure 15:**
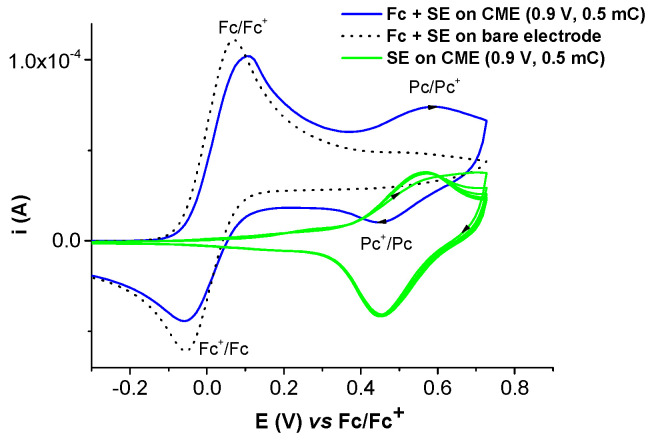
CV curves (0.1 V/s) of Ni(II)**L**-CMEs (prepared via CPE at 0.9 V and using an electrical charge of 0.5 mC in a solution of Ni(II)**L** (0.11 mM) in 0.1 M TBAP/CH_3_CN) transferred into 0.1 M TBAP/CH_3_CN (green line)—5 successive CV curves—and into ferrocene solution (1 mM) in 0.1 M TBAP/CH_3_CN (blue line) vs. bare electrode (dashed line).

Several chronoamperograms obtained during the preparation of CMEs via CPE are shown in Figure 12a and Figure 13a. They are reproducible, as shown in Figure S2a. The modified electrodes obtained via CPE, transferred into the TBAP/CH_3_CN and ferrocene solutions, have their CV curves represented in Figure 12b, Figure 13b, Figure 14, Figure 15 and Figure S2b.

The electrochemical characterization of the modified electrodes in 0.1 TBAP/CH_3_CN without or with ferrocene solution in the supporting electrolyte is shown in Figure 14 and Figure 15.

### 2.3. Conductivity Studies

The molar conductivity (Λm) value for the H_2_**L** and Ni(II)**L** metal complex measured in dimethylsulphoxide (DMSO) was 0 in the range of 0–45 ohm^−1^ cm^2^ mol^−1^.

### 2.4. UV-Vis Studies

**Figure 16:**
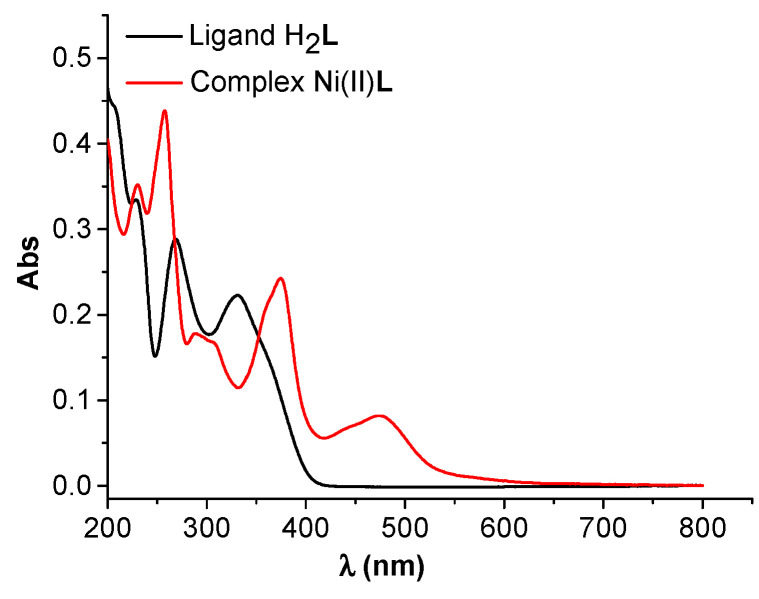
UV-Vis spectra of H_2_**L** (2 mM) and complex Ni(II)**L** (1 mM) in acetonitrile.

**Figure 17:**
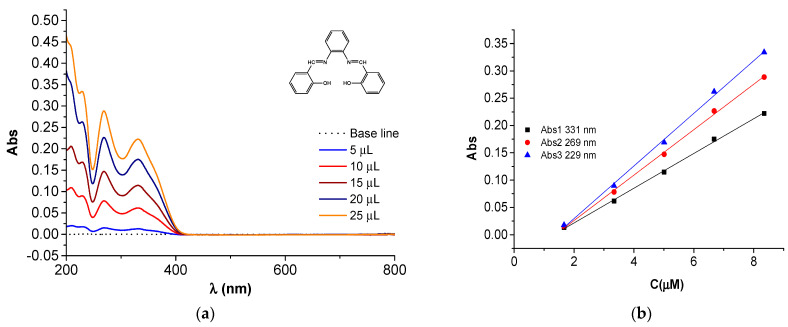
(**a**) UV-Vis spectra of the H_2_**L** in acetonitrile for different amounts of solution (2 mM) in acetonitrile; (**b**) calibration curves at different wavelengths.

**Figure 18:**
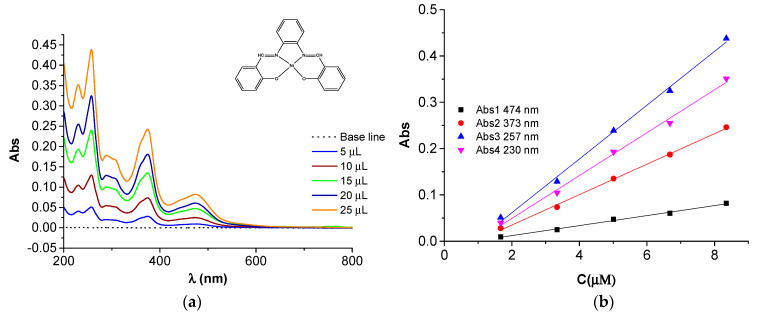
(**a**) UV-Vis spectra of the Ni(II)**L** in acetonitrile for different amounts of solution (1 mM) in acetonitrile; (**b**) calibration curves at different wavelengths.

The UV-visible spectra of the H_2_**L** ligand and Ni(II)**L** complex are shown in Figure 16. Their spectra were recorded in acetonitrile at different concentrations (Figure 17 and Figure 18) for H_2_**L** and Ni(II)**L** complex, respectively. The main characteristics are shown in Table 1.

### 2.5. Thermogravimetric Studies

**Figure 19:**
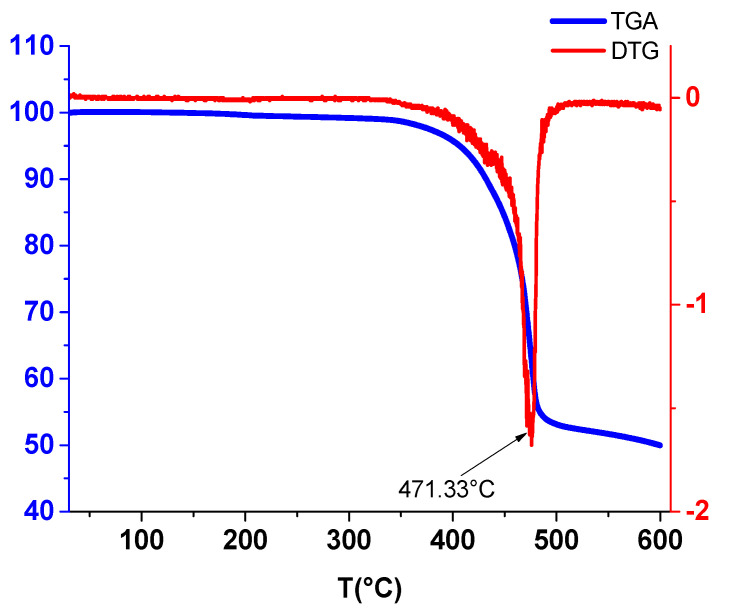
TGA/DTG of Ni(II)**L** (M = 374.06); M(NiO) = 74.69.

The thermal study of Ni(II)**L** was carried out. The TGA/DTG curves are shown in Figure 19.

### 2.6. Antimicrobial Activity Studies

The results are presented in Table 2. The antimicrobial activities of all of the compounds were studied against four bacterial cultures: Gram-positive (*B. cereus*, *S. aureus*) and Gram-negative (*S. typhi*, *E. coli*). In addition, the antifungal activities of these compounds were studied against three fungal cultures of yeast (*C. albicans*), mold (*A. niger*), and fungus (*A. Carbonarius*). The standard antibacterial (Chloramphenicol) and antifungal (Nystatin) drugs were used as positive controls. The in vitro antibacterial and antifungal activity of the precursors (orthophenylenediamine—OPD and salicylaldehyde—SA) and control drugs were evaluated at a concentration of 40 mg/mL, while the H_2_**L** ligand and Ni(II)**L** complex were tested at three different concentrations: 20, 30, and 40 mg/mL, using the disc diffusion method [26]. The susceptibility of the microorganisms to these compounds was evaluated by measuring the clear inhibition zones caused by the samples against the selected strains of bacteria and fungi and under identical experimental conditions. The margin of activity is a diameter ≥ 6 mm.

### 2.7. Antioxidant Activity Studies

**Figure 20:**
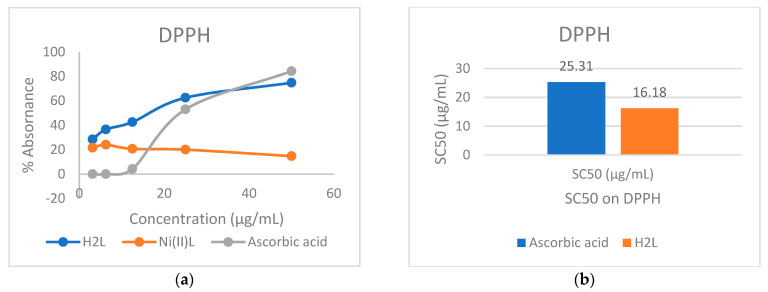
(**a**) Dependences of free radical scavenging activity on DPPH concentration; (**b**) SC50 on DPPH of the ascorbic acid (AA) and the ligand H_2_**L**.

The antioxidant (radical scavenging) activity on 2,2-diphenyl-1-picryhydrazyl (DPPH) of H_2_**L** and Ni(II)**L** was investigated at different DPPH concentrations, as shown in Figure 20a, and compared with that of ascorbic acid (AA). The SC50 (concentration causing 50% inhibition) for H_2_**L**, Ni(II)**L,** and AA was evaluated, as seen in Figure 20b.

## 3. Discussion

The use of differential pulse voltammetry (DPV) allowed for clear evidence of the processes occurring during the oxidation or reduction of the investigated compounds, which are seen when scanning toward positive or negative potentials. Cyclic voltammetry (CV) in different scan domains and at variable scan rates allow one to establish the degree of reversibility of the evidenced processes, and rotating disc electrode voltammetry (RDE) helps to establish the potentials favorable for polymeric film formation.

From the comparison of the anodic and cathodic curves obtained via DPV and CV in the same potential ranges from Figure 2, it can be found that the DPV anodic peaks of the H_2_**L** ligand are situated at Epa1 = +0.766 V and Epa2 = +1.517 V, and the two cathodic peaks are situated at Epc1 = −1.898 V and Epc2 = −2.231 V. They correspond to the oxidation/reduction processes of H_2_**L**. The CVs of H_2_**L** (Figure 2) are in agreement with the DPV curves and show two anodic peaks at +0.820 V (a1) and +1.623 V (a2), corresponding to the successive two-electron transfer processes of H_2_**L**. The currents increase with the ligand concentration. These results agree with those obtained for differently substituted compounds [27,28]. In the cathodic CV scans, two reduction waves were noticed at −1.958 V (c1) and −2.698 V (c2), corresponding to the successive reduction of the azomethine group (>C=N) of H_2_**L** [27,28,29,30].

Figure 3 shows the first cycles of the CV curves in different anodic and cathodic scan domains. The anodic scans put into evidence more irreversible processes than the cathodic ones which are quasi-reversible (two pairs of peaks: c1/c1′ and c2/c2′). However, when the scan rate increases, an a1′ peak (corresponding to the a1 peak) becomes visible in the return scans.

Figure 4a shows the increase in the currents with the scan rate, and Figure 4b shows the linear variation in the peak currents for processes a1, a1′, a2, and c1, respectively, with the square root of the scan rate. 

When the CV scan rate increases, the dependences of the peak potential (Ep) on the scan rate are linear, with slopes in mV/decade of 62 for a1, 29 for a2, 22 for a1′, and –44 for c1 (Table S5). The dependences of the peak currents (ip) for each process on the square root of the scan rate are also linear with slopes of 3.4 × 10^−5^, 10.1 × 10^−5^, −0.9 × 10^−5^, and −14.8 × 10^−5^ A∙V^−1^∙s^1/2^, respectively (Table S6). These results show that the electrode processes correspond to controlled diffusion quasi-reversible systems [31].

The RDE curves (Figure 5) obtained for different solutions of H_2_**L** show regular behavior (with the limiting current depending on the H_2_**L** concentration and on the rotation rate of the electrode) in the cathodic range of potential corresponding to peak c1. The anodic RDE curves agree with the passivation of the GC electrode, as the currents are close to the background in the entire range of anodic potentials. This behavior could be explained by the film formation at anodic potentials that was checked using all of the electrochemical methods available in our laboratory.

The CV study of Ni(II)**L** showed a well-defined anodic wave at +0.714 V, corresponding to one-electron oxidation which can be attributed to the Ni(II)→Ni(III) oxidation process, as shown in previous works [32,33,34]. The anodic peak at +1.525 V due to the electron transfer reaction centered on the ligand may be related to the oxidation of **L** from Ni(II)**L**. In the cathodic scan, the CVs of the complex show two reduction waves at −1.817 V and −2.466 V, which may be related to the irreversible reduction of the ligand [33] in the complex. On the cyclic voltammogram of the Ni(II)**L** complex, the increases in the peak currents for processes a1, a1′, and c1, respectively, are linear with the square root of the scan rate (Figure 8b), suggesting that the electrode processes are controlled via diffusion [35,36].

The RDE curves obtained for different solutions of Ni(II)**L** at a constant rotation rate at different concentrations are regular in the cathodic domain at potentials corresponding to peak c1. The RDE curves in the anodic domain indicate the formation of conductive films at potentials close to peak a1 and insulating films at potentials higher than 1.5 V.

The electrochemical behavior of Ni(II)**L** was carefully investigated via CV, DPV, and RDE (Figure 6, Figure 7, Figure 8 and Figure 9) to understand the formation of blue films, which were noticed at the end of the anodic potential scans.

The plots of Ep vs. log v indicate the differences between the films prepared via electropolymerization, performed during scanning at different potentials (Table S9). The slopes of Epa1 vs. log v are of 3 mV/ decade, 74 mV/ decade, and 113 mV/ decade for the anodic scan limit (*vs* RE) of 0.7 V, 0.9 V, and 1.12 V, respectively. That means the electropolymerization occurs selectively at 0.7 V (the foot of the wave) as the a1 peak potential is quite constant, and less selectively at higher polymerization limits of 0.9 V and 1.12 V as the a1 peak potential vary with 74 mV/decade and 113 mV/decade, respectively. The plots of Epa1′ vs. log v indicate the differences between the films prepared via electropolymerization performed during the scanning at different potentials (Table S9). The slopes are 0 mV/ decade, 24 mV/ decade, and 23 mV/ decade for an anodic scan limit of 0.7 V, 0.9 V, and 1.12 V, respectively. This means that the electropolymerization leads to films of the same type when the scan limit is 0.7 V, 0.9 V, and 1.12 V.

The plots of the currents on the square root of the scan rate (ip vs. v^1/2^) for peaks a1 and a1′ (Table S10) also vary. For the peak a1, they are 2 × 10^−4^ A × (V/s)^−1/2^ and 2.17 × 10^−4^ A × (V/s)^−1/2^ for the anodic scan limits of 0.9 V and 1.12 V, respectively. These values mean similar rates of polymerization occurring at these limits of potential. For the peak a1′, the dependences ip vs. v^1/2^ have slopes of −0.08 × 10^−4^ A × (V/s)^−1/2^, −0.664 × 10^−4^ A × (V/s)^−1/2^, and −1.095 × 10^−4^ A (V/s)^−1/2^ for an anodic scan limit of 0.7 V, 0.9 V, and 1.12 V, respectively (Table S10). This indicates slow kinetics at 0.7 V, and a rapid one at the potentials of 0.9 V and 1.12 V, where the polymerization rates were close.

The study of film formation via CV showed the evolution of the curves in the first five successive cycles (Figure 10). The analysis of the cycles obtained in the potential range (0–1.1 V) shows that the first anodic peak a1 is not exclusively from the Ni(II)**L** oxidation to Ni(III)**L,** as it is a large peak. The possible processes could also be the oxidation of the Ni(II)-phenoxy radical complex into the Ni(II)-bis-phenoxy radical complex followed by chemical polymerization [37,38,39,40,41]. In the first reverse scan, peak a1′ appears (at a lower potential than a1). In the second cycle (dashed line in Figure 10), it is like a shoulder. In the following cycles, this shoulder goes to a peak (a0), which increases continuously during the scanning. A similar increase occurs for peak a1′ during scanning. Peak a1 is almost constant with cycling, while the currents of the CV curves between 0.8 V and 1.1 V in the direct scans slowly increase. This behavior is consistent with more oxidation processes centered either on the ligand or on Ni(II) ion oxidation, in agreement with other studies [41]. This behavior is valid for potential domains that do not exceed 1.5 V.

The noticed film formed via scanning at potentials more positive than 1.1 V (Figure 11) is insulating as a1′ peak disappears from the second cycle and the currents for the a1 and a2 peaks decrease during the cycling process.

The chemically modified electrodes (CMEs) thus obtained proved to be electroactive in the studied supporting electrolyte solutions (TBAP/CH_3_CN) and in ferrocene (Fc) in the supporting electrolyte solutions. The curves of the CMEs recorded in Fc were compared with those on the bare electrode.

The CMEs prepared via CPE were examined using chronoamperometry during the preparation. The chronoamperograms were in agreement with the applied potentials. The CMEs prepared at different potentials and using the same amount of electrical charge such as those from Figure 12 after transfer in Fc solution reveal an increase in Fc activity at all preparation potentials. The anodic current is higher than on the bare electrode on all CMEs, but especially on those prepared at 0.9 V and 1.1 V. This increase can be attributed to the redox couple of Ni(II)/Ni(III) from the CMEs. There is a shift in potential toward positive potentials for all of the CMEs.

The CMEs prepared at the same potential and using different amounts of electrical charges such as those from Figure 13 after transfer in Fc solution reveal a pronounced increase in Fc activity which increases with the film thickness. The anode current on the CMEs increases with the electrical charge used in the synthesis, and the anode potential shifts to increasingly positive values with increasing charge.

The characterization of Ni(II)**L**-CMEs prepared via CPE in a solution of Ni(II)**L** in the supporting electrolyte (0.1 M TBAP/CH_3_CN) via DPV and CV (Figure 14) put into evidence the signal characteristic of the formed film. The successive CV curves indicate the stability of the formed film. The DPV appears at about 0.5 V, indicating the presence of Ni(II)/Ni(III) in a polymer film. The pair of peaks corresponds to the reduced and oxidated forms of the polymer (Pc/Pc+), which appears at about 0.5 V.

The CV curves of Ni(II)**L**-CMEs recorded in ferrocene solution in the supporting electrolyte (Figure 15) show the presence of both couples Fc/Fc+ and Pc/Pc+.

The value of the molar conductivity indicates that the Ni(II)**L** complex is non-electrolytic in DMSO, contrary to its behavior in other solvents [42], since for 1:1 electrolytes, a value below 100 ohm^−1^·cm^2^·mol^−1^ is often expected.

From the UV-visible spectrum of H_2_**L** (Figure 16 and Figure 17), a broad absorption band between 269 nm (37,175 cm^−1^) and 331 nm (30,215 cm^−1^) can be seen, corresponding to the π→π* and n→π* transition of the azomethine group (>C=N) which is responsible for the orange-yellow coloration of the ligand.

The UV-visible spectrum of the Ni(II)**L** complex is shown in Figure 16 and Figure 18. Its spectrum shows two bands at 257 (38,911 cm^−1^) and 374 nm (26,738 cm^−1^) attributed to the π–π^∗^ and n–π^∗^ transitions, and one band in the visible region around 474 nm (21,097 cm^−1^) assigned to the ^1^A_1g_→^1^B_1g_ transition, responsible for the coloration of this complex and in agreement with square planar geometry. The electronic data for H_2_**L** and Ni(II)**L** are summarized in Table 1. These data obtained at the maximum wavelengths made it possible to establish linear dependences of absorbance peaks on the concentrations of the solutions with slopes 0.0416 M^−1^ and 0.0318 M^−1^ for H_2_**L** at 269 nm and 331 nm. For Ni(II)**L,** the slopes are 0.0581 M^−1^, 0.0329 M^−1^, and 0.0108 M^−1^ at 257 nm, 373 nm, and 474 nm. This characterization confirms that the ligand acts as a quadridentate dianionic ligand (through the disappearance of the vibrational band for the hydroxyl group). The Schiff base linkage was also observed to be shifted by ∆υ = 8 cm^−1^ upon complexation with the Ni(II) ion.

The thermal study of Ni(II)**L** was carried out according to the model described by El-Sawaf et al. following the evaluation of the percentages of mass loss according to the temperature ranges and the nature of the phenomena (endothermic or exothermic). The TGA/DTG curves of the Ni(II)**L** are in agreement with the literature data [43,44] and show its thermal decomposition occurring in a single step, while that of H_2_**L** occurs in two steps [44]. The decomposition of Ni(II)**L** takes place over a temperature range and is coupled with an endothermic phenomenon on the DTG curve at 471.33 °C, corresponding to a mass loss of approximately 42% (calculated as 48%), associated with the elimination of [2C_6_H_4_ + H_2_ + N_2_]. Above 500 °C, the final residue can be attributed to [C_7_H_5_ + CO + NiO], with a mass loss percentage of about 50% (calculated as 51%).

In the study of antimicrobial activity, all of the compounds and reference drug controls showed activities against the bacteria and fungi. The red color in Table 2 indicates the values of the diameters of the zones of inhibition of the compounds, which are very active against the bacteria and fungi, while the green and orange colors indicate the values of the diameters of the zones of inhibition of the compounds with medium (under 14) and low (under 9) activity, respectively. The black color indicates very low activity.

The Schiff base (H_2_**L**) is weakly active on all microorganisms, with an inhibition zone diameter around 6 mm. On the contrary, the Ni(II)**L** exhibited higher activity compared to that of H_2_**L** on the bacteria: *E. coli* and *S. tiphy* were at 40 mg/mL and *S. tiphy* and *S. aureus* were at 20 mg/mL as the inhibition zone diameter ranged between 10 and 18 mm. The Ni(II)**L** complex also shows antifungal action, which is manifested in the concentration of 30 mg/mL for *A. Carbonarius* and 20 mg/mL for *A. niger* and *A. Carbonarius*. It is also noteworthy that the antimicrobial or antifungal effect of Ni(II)**L** is higher at the lower concentration (20 mg/mL) than at the bigger concentration (40 mg/mL).

The antimicrobial activities of the compounds at different concentrations are given in Table 2 and are in agreement with other recent works in the literature [45,46]. The minimum concentration to inhibit bacterial growth by 90% (MIC_90_ value) for the antibacterial activity of the H_2_**L** ligand and the Ni(II)**L** complex was evaluated by Baecker et al. [45]. The ligand did not inhibit bacterial growth even at the highest concentration of 100 µg/mL. The complex was shown to be inactive against Gram-negative bacteria *E. coli* and *P. aeruginosa* at the same concentration [45]. However, the Ni(II)**L** complex that we obtained showed antibacterial inhibitory properties at a concentration of 40 mg/mL on *E. coli* and *S. tiphy* with diameters of zones of inhibition of 10 and 11 mm. The antibacterial potential of a similar ligand substituted by a methyl and ethoxy group and its Ni(II) complex was evaluated through in vitro screening by Kargar et al. in solutions of a concentration of 1 mg/mL [46]. Another ligand similar to H_2_**L** was found to be more active on *E. coli* and *S. aureus* with diameters of zones of inhibition of 10 and 21 mm, while its Ni(II) complex showed improved antibacterial activity compared to the free ligand, with diameters of zones of inhibition of 15 and 25 mm [46].

This enhancement of the activity of the metal complex compared to the ligand was explained by the chelation effect, which reduced the polarity of the metal ion. An increase in π-electron delocalization leads to an increase in liposolubility, which is an important fact controlling antimicrobial activity, because an enhancement in lipophilicity increases the concentration of complexes in the lipid membrane, limiting the microorganisms’ multiplication [47,48].

It is evident from the results in Figure 20 that the free radical scavenging activities of the Schiff base ligand H_2_**L** and the Ni(II)**L** complex are concentration-dependent, as is the case for other compounds [26,49]. At low DPPH concentrations, the ligand H_2_**L** and Ni(II)**L** complex have higher free radical scavenging activity than AA (reference antioxidant), while at a higher concentration (over 35 µg /mL), they are lower than AA. The SC50 (concentration causing 50% inhibition) for Ni(II)**L** cannot be evaluated. For AA and H_2_**L** SC50 they are 25.31 and 16.18 µg/mL, respectively, suggesting that the ligand H_2_**L** is moderately active.

## 4. Materials and Methods

### 4.1. Reactants

All reagents were purchased from Riedel-de Haen company, Acros Organics, and used as received. Acetonitrile (CH_3_CN) and tetra-n-butylammonium perchlorate (TBAP) from Sigma-Aldrich (Darmstadt, Germany) were used as received. Ethanol, methanol, tetrahydrofurane (THF) from Riedel-de Haen, and dimethylsulfoxide (DMSO) from Acros Organics used as solvents are commercially available and were used without further purification. Antioxidant activity was evaluated using a methanolic solution of 2,2-diphenyl-1-picrylhydrazyl (DPPH) and ascorbic acid (AA) from Sigma-Aldrich (Saint Quentin Fallavier, France).

Antibacterial activities were carried out against *E. coli* ATCC25922, *B. cereus* ATCC11966, *S. typhi*, and *S. aureus* SR196, and antifungal activities were carried out against *C. albicans*, *A. niger* NRRL612, and *A. carbonarius* NRRL368. The tested bacterial strain of *Escherichia coli* ATCC25922 was from the Pasteur Center in Cameroon and the other tested strains were from the BEI Resources strain collection.

### 4.2. Apparata

^1^H and ^13^C-NMR spectra were recorded using a Gemini 300 BB spectrometer operating at 300 MHz for ^1^H and 75 MHz for ^13^C in DMSO-d_6_, using TMS as the internal reference. Varian 310–MS LC/MS/MS triple quadrupole mass spectrometer fitted with an electrospray ionization interface (ESI) was used. Air was used as the drying gas at a pressure of 19 psi and temperature according to the experiment. The nebulizing gas was nitrogen of 40 psi for positive ionization and air of 55 psi for negative ionization. The needle voltage was established to the potential 5000 V for positive ionization and −4500 V for negative ionization. The substances were solubilized in DMSO and diluted with MeOH. The solution thus obtained was injected directly into the interface using a syringe pump Harvard 11PLUS, with a 0.010 mL/min flow. Thus, the protonated or deprotonated molecular ion obtained was selected using the first quadrupole. In the second quadrupole, the protonated or deprotonated molecular ion was fragmented via collision with inert gas (argon) to a pressure of 1.5 mTorr. Fragments were analyzed using the third quadrupole. Prior to these experiments, the tuning of the mass spectrometer was performed using PPG for both positive and negative.

Infrared spectra were recorded using a Bruker FT-IR tensor 27 spectrometer directly on small samples of the compounds in the range of 4000–400 cm^−1^.

UV-Vis spectra were recorded using a JASCO V-670 spectrometer in 1 cm path-length quartz cuvettes.

Thermogravimetric analysis of the complexes was carried out using a Perkin-Elmer Pyris 6 TGA in a closed perforated aluminum pan simultaneous thermal analyzer.

Conductometric measurements were made on Digital Conductivity model Labtech.

Electrochemical investigations were performed using the PGSTAT 12 AUTOLAB potentiostat, to which three-compartment cells were coupled. The working electrode (WE) was a glassy carbon (GC) disk with a diameter of 3 mm (Metrohm, Herisau, Switzerland), bare or modified. Ag/10 mmol∙L^−1^ TBAP/CH_3_CN (0.1 M) was used as the reference electrode (RE), while a platinum wire was used as an auxiliary electrode (AE). The assembly of RE, WE, and AE in line was connected to an AUTOLAB potentiostat, controlled by NOVA software (Version 2022). All potentials were referred to the ferrocene/ferrocenium couple. For film formation experiments, a transfer cell with a GC-modified electrode as WE, a Pt wire as AE, and Ag/AgCl and KCL 3M as RE were used.

In vitro antibacterial and antifungal activities of the studied compounds were tested using Brain Heart Infusion agar solidified medium for the bacterial strains and Sabouraud Dextrose Agar (SDA) for the fungal strains.

Antioxidant activity was evaluated using a methanolic solution of 2,2-diphenyl-1-picrylhydrazyl (DPPH) and ascorbic acid (AA) using the Tecan Infinite M200 Spectrophotometer.

### 4.3. Procedures

#### 4.3.1. Synthesis of Schiff Base *N*,*N*’-Bis-(2-hydroxybenzylidene)-benzene-1,2-diamine (H_2_L)

The Schiff base ligand was prepared according to the literature method using 1:2 molar reactant ratios of orthophenylenediamine and salicylaldehyde [50]. Salicylaldehyde (20 mmol, 2.08 mL) was dissolved in 20 mL of ethanol in a 100 mL beaker at room temperature. A solution of orthophenylenediamine (10 mmol, 1.08 g) dissolved in 20 mL of ethanol was added dropwise to the salicylaldehyde solution while stirring. The mixture was heated under reflux for 3 h in a water bath at a temperature of 80 °C and cooled to room temperature. The yellowish-orange crystalline powder was obtained, which was filtered and dried. This resulted in 2.682 g of ligand H_2_L (yield 85%) which was characterized via NMR [51,52,53], UV-Vis [54,55,56], and IR [57,58].

Ligand H_2_**L**: M.p: 165 °C. ^1^H-NMR (CDCl_3_, δ ppm): 13.20 (s, 2H, 2OH), 8.92 (s, 2H, H1, H1′), 7.67 (dd, 7.7; 1.8, 2H, H7, H7′), 7.50–7.35 (m, 6H, H5, H5′, H10-H13), 7.04 (d, 7.9, 2H, H4, H4′), 6.92 (m, 7.6,1.2, 2H, H-6). ^13^C-NMR (CDCl_3_, δ ppm): 163.86 (C-1, C-1′), 161.49 (C-3, C-3′), 142.70 (C9, C14, C9′, C14′), 133.50 (C7, C7′), 132.47 (C5, C5′), 127.83 (C11,12), 119.87 (C10, C13), 119.36 (C2, C2′), 119.10 (C4(6), C4′ (6′)), 117.69 (C-6(4), C-6′ (4′)). Anal. Calc. for C_20_H_16_N_2_O_2_ (316.36): C, 75.93; H, 5.10; N, 8.86; O, 10.11. Selected ν IR data (solid ATR, cm^−1^): 3054 (OH), 1611vi (C=N), 1560i (C=C), 1476i, 1375 (C-N), 1275 m (C-O), 1185 m, 748v + 635/i.

#### 4.3.2. Synthesis of Ni(II)L Complex

The preparation of the nickel(II) complex illustrates this synthesis. A solution of *N*,*N*’-Bis-(2-hydroxybenzylidene)-benzene-1,2-diamine (1.5 mmol, 0.475 g) in 20 mL ethanol was gradually added with stirring to 20 mL ethanol solution of nickel(II) chlorite hexahydrate (1.5 mmol, 0.255 g). The reaction mixture was kept in the water bath for refluxing for about 2 h. The colored complex separated out and the product was filtered and dried at room temperature. Ni(II)L: the complex was obtained as a brick red solid. Crystals suitable for single crystal analysis were obtained from recrystallization in chloroform/ethanol. Yield: 0.529 g (94%). The complexes were characterized via NMR [43,51,57,59], MS [60,61,62,63,64], IR [65,66,67], UV-Vis [68,69], conductometry [42,70], and thermogravimetry [44,71,72].

Ni(II)**L**: M.p: ˃360 °C. ^1^H-NMR (DMSO-d6, δ ppm): 8.90 (s, 2H, H1, H1′), 8.14 (m, 2H, H11, H12), 7.62 (dd, 1.8, 7.9, 2H, H-7), 7.3–7.4 (m, 4H, H10, H13, H5, H5′ ), 6.90 (d,8.2, 2H, H4, H4′), 6.68 (t, 7.0, 2H, H6, H6′). ^13^C-NMR (DMSO-d6, δ ppm): 165.36 (C1, C1′), 156.63 (C3, C3′), 142.39 (C9(14), C9 (14)), 135.37 (C7, C7′), 134.31 (C5, C5′), 127.75 (C11(12), C11′(12′)), 120.37 (C10(13), C10′ (13′)), 120.25 (C2, C2′), 116.43 (C6, C6′), 116.24 (C4, C4′), 115.42 (C8, C8′). Anal. Calc. for C_20_H_14_N_2_O_2_Ni (374.06): C, 64.05; H, 4.30; N, 7.47; Ni, 15.65; O, 8.53. Selected ν IR data (solid ATR, cm^−1^): 1603 (C=N), 1516 (C=C), (C-N), (C-O), 543(Ni-N), 459(Ni-O).

#### 4.3.3. Procedure for Voltammetric Investigations

Electrochemical experiments for ligand characterization and the preparation of modified electrodes were performed via cyclic voltammetry (CV), differential pulse voltammetry (DPV), and rotating disk electrode voltammetry (RDE). For recording CV curves, WE, RE, and CE were immersed in the electrochemical cell containing the supporting electrolyte (0.1 M TPAP/CH_3_CN). The solutions of the compounds (1.0 × 10^−3^ M) were prepared, and the cyclic voltammograms were recorded at different scan rates from stationary potential to −3 V (for cathodic scans) or +3 V (for anodic scans). DPV curves were recorded at 0.01 V/s and RDE experiments were performed at 0.01 V/s with rotation rates between 500 and 1500 rpm. Before each experiment, the glassy carbon electrode was properly cleaned by polishing it with diamond paste (0.25 µm). The solutions of study in TBAP/CH_3_CN were purged of oxygen by bubbling dry nitrogen gas for 15 min, and they were then covered with the same gas during the experiments. At the end of the experiments, the potential referred to the potential of the ferrocene/ferrocenium redox couple (Fc/Fc+).

#### 4.3.4. Procedure for Film Formation

The film formation was performed via electrochemistry either through scanning the potential or through controlled potential electrolysis (CPE) followed by chronoamperometry in millimolar solutions of Ni(II)**L** in 0.1 M TBAP in acetonitrile, on standard glassy carbon (GC) electrodes. Then, the chemically modified electrodes (CMEs) were rinsed and transferred to another electrolyte in order to characterize the deposition. The electroactivity of CMEs was studied in the supporting electrolyte (0.1 M TPAP/CH_3_CN) and in ferrocene solution in the supporting electrolyte via CV and DPV. Each CME was washed with acetonitrile and introduced into a transfer cell containing 0.1 M TPAP/CH_3_CN first, and then a solution of ferrocene in 0.1 M TPAP/CH_3_CN. Their CV curves were recorded. Thus, the CV curve evolution was followed, and the first and second CV curves were compared.

When the GC electrode was modified via scanning, five CV curves were recorded successively, usually. For this purpose, complex solutions of different concentrations were used.

#### 4.3.5. Procedure for Conductometric, UV-Vis, Thermogravimetric, Antimicrobial Activity, and Antioxidant Activity Studies

The complexes were dissolved in DMSO/THF mixtures, and the molar conductivity of their solution at room temperature was measured.

UV-Vis spectra were recorded in acetonitrile between 200 and 800 nm in freshly dried acetonitrile.

Thermogravimetric analysis of the complexes was carried out at a 10 °C/min heating rate up to 600 °C under a nitrogen atmosphere.

Antibacterial and antifungal activities of the ligand H_2_**L** and complex at different concentrations (20, 30, and 40 mg/mL) were carried out. The disks’ impregnated test products were deposited on the surface of Petri dishes seeded and incubated aerobically at 37 °C for bacterial strains and fungal strains for 24 h and 3 days, respectively. The diameter (mm) of the area of inhibition around each disc was measured after 24 h.

Antioxidant activity was evaluated via the spectrophotometric method at 517 nm using DPPH. To a methanolic solution of DPPH (0.01 mmol) and ascorbic acid (AA), ligand H_2_**L** and Ni(II)**L** complex were added separately at different concentrations (200; 100; 50; 25; 12.5; 6.25; 3.125; 1.56; and 0.78 μg/mL), and an equal amount of methanol (2 mL) was added as the control. After 30 min at 30 °C, absorbance was measured. The activity was compared with that of AA which was used as a standard antioxidant. The percentage of free radical scavenging (FRSA) was calculated using Equation (1), where Ao is the absorbance of DPPH without analyte (blank), and As is the absorbance of samples with complex or ligand. The % inhibitions were plotted against the respective concentrations used and from the graph, the concentrations causing 50% inhibition SC50 values were calculated.

FRSA = (Ao − As)/(Ao × 100)(1)

## 5. Conclusions

This study concerns a deep exploration of the free Schiff base *N*,*N*’-Bis-(2-hydroxybenzylidene)-benzene-1,2-diamine (H_2_**L**) and its Ni(II) complex (Ni(II)**L**, to find broader applications not yet explored. H_2_**L** and Ni(II)**L** were synthesized and confirmed using spectroscopic methods (NMR, FT-IR, UV-Vis, electrospray ionization mass spectroscopy, thermogravimetric analysis, and molar conductance measurements). These methods also proved that H_2_**L** acts as a tetradentate ligand and coordinates to the central metal through nitrogen and oxygen atoms.

The electrochemical behavior of H_2_**L** and Ni(II)**L** was investigated in anodic and cathodic scan domains using DPV, CV, and RDE techniques to find new details for future applications. Ni(II)**L** exhibited an irreversible electron transfer and redox-diffusion-controlled processes in the anodic scans. Anodic electropolymerization was used to form chemically modified electrodes (CMEs). The results of these electropolymerization processes were blue films on the electrode surfaces in the entire potential range studied. They were obtained either by scanning the potential in the anodic domain, or via controlled potential electrolysis (CPE). The chronoamperometry indicated good reproducibility in film preparation via CPE. The CMEs’ electrochemical tests showed excellent stability and an increase in current intensities after the transfer in ferrocene solutions.

H_2_**L** and Ni(II)**L** were tested for their antimicrobial activity against the bacteria and fungi at different concentrations, showing good activities on several Gram-negative bacteria and also an antifungal effect.

## Figures and Tables

**Table 1:** Electronic spectral data in acetonitrile for H_2_**L** and Ni(II)**L** and their calibration curves.

Compound	Band Maxima (cm^−1^)	Wavelength (nm)	Assignment	Calibration Equation/Figure (C in µM)
H_2_L	37,175	269	n→π*	Abs2 = −0.0573 + 0.0416·C/Figure 17b
	30,215	331	π→π*	Abs1 = −0.0422 + 0.0318·C/Figure 17b
Ni(II)L	38,911	257	π→π*	Abs3 = −0.0546 + 0.0581·C/Figure 18b
	26,738	373	n→π*	Abs2 = −0.0308+ 0.0329·C/Figure 18b
	21,097	474	^1^A_1g_→^1^B_1g_	Abs1 = −0.0095 + 0.0108·C/Figure 18b

**Table 2:** Diameter (in mm) of inhibition zones of H_2_**L** and Ni(II)**L** and of different drugs against bacteria and fungi at different concentrations.

Compound	Bacteria *				Fungi **		
	Gram-Positive		Gram-Negative		Yeast	Mold	Fungus
	*B. cereus*	*S. aureus*	*S. typhi*	*E. coli*	*C. albicans*	*A. niger*	*A. carbonarius*
**40 mg/mL**							
Chloramphenicol	16.00 ± 2.00	22. 00 ± 3.00	19.00 ± 1.00	18.00 ± 2.00	/	/	/
Nystatine	/	/	/	/	19.00 ± 2.00	23.00 ± 5.00	27.00 ± 3.00
OPD ***	7.00 ± 0.00	14.00 ± 4.00	22.00 ± 4.00	17.00 ± 1.00	9.00 ± 2.00	6.00 ± 0.00	17.00 ± 3.00
SA ****	6.00 ± 0.00	6.00 ± 0.00	6.00 ± 0.00	6.00 ± 0.00	6.00 ± 0.00	6.00 ± 0.00	6.00 ± 0.00
H_2_L	6.00 ± 0.00	6.00 ± 0.00	6.00 ± 0.00	6.00 ± 0.00	6.00 ± 0.00	6.00 ± 0.00	6.00 ± 0.00
Ni(II)L	6.00 ± 0.00	6.00 ± 0.00	11.00 ± 0.00	10.00 ± 0.00	6.00 ± 0.00	6.00 ± 0.00	6.00 ± 0.00
**30 mg/mL**							
H_2_L	6.00 ± 0.00	6.00 ± 0.00	6.00 ± 0.00	6.00 ± 0.00	6.00 ± 0.00	6.00 ± 0.00	6.00 ± 0.00
Ni(II)L	6.00 ± 0.00	6.00 ± 0.00	6.00 ± 0.00	6.00 ± 0.00	6.00 ± 0.00	6.00 ± 0.00	18.00 ± 0.00
**20 mg/mL**							
H_2_L	6.00 ± 0.00	6.00 ± 0.00	6.00 ± 0.00	6.00 ± 0.00	6.00 ± 0.00	6.00 ± 0.00	6.00 ± 0.00
Ni(II)L	6.00 ± 0.00	7.00 ± 0.00	15.00 ± 0.00	6.00 ± 0.00	6.00 ± 0.00	11.00 ± 0.00	14.00 ± 9.00

* Bacillus cereus ATCC1966 (B. cereus); Staphylococcus aureus (S. aureus); Salmonella tiphy SR196 (S. typhi); Escherichia coli ATCC25922 (E. coli); ** Candida albicans (C. albicans); Aspergillus niger NRRL612 (A. niger); Aspergillus carbonarius NRRL368 (A. carbonarius); *** Orthophenylenediamine (OPD); **** Salicylaldehyde (SA).

## Data Availability

Not applicable.

## Supplementary Materials

The following supporting information can be downloaded at https://www.mdpi.com/article/10.3390/molecules28145464/s1, Figure S1: Anodic and cathodic DPV and CV 0.1 V/s curves on GC of H_2_**L** and Ni(II)**L** complex superimposed at 1 mM concentrations in 0.1 M TBAP/CH_3_CN; Figure S2: (a) Chronoamperograms during the preparation of Ni(II)**L**-CMEs in 0.4 mM solution of Ni(II)L in 0.1 M TBAP/CH_3_CN, via CPE at different charges; (b) CV curves after their transfer in ferrocene solution at 1 mM in 0.1 M TBAP/CH_3_CN vs. bare electrode; Figure S3: (a) Anodic cyclic voltammograms with anodic scan limits (vs. RE) of 0.7 V (a), 0.9 V (b), and 1.13 V (c) at different scan rates for solution of [Ni(II)**L**] = 0.4 mM in 0.1 M TBAP/CH3CN; (b) linear dependences of a1 and a1′ peak currents on the square root of the scan rate, and linear dependences of a1 and a1′ peak potentials on the logarithm of the scan rate. Table S1: Potential and current of the peaks in anodic and cathodic scans from CV, DPV, and half-wave potential from RDE experiments for H_2_**L**; Table S2: Influence of the scan rate on the potential and current of the anodic and cathodic peaks in CV scans for H_2_**L**; Table S3: Potential and current of the anodic and cathodic peaks in CV and DPV scans, and half-wave potential from RDE experiments for Ni(II)**L** complex; Table S4: Influence of the scan rate on the potential and current of a1 and a1′ peaks in anodic scans and c1 peak in cathodic scans in CV experiments for Ni(II)**L** complex; Table S5: Equations for the dependences of peak potential and peak current on the scan rate v and their correlation coefficients for a1, a1′, a2, and c1 peaks for H_2_**L**, according to Figure 4a.; Table S6: Equations for the dependences of peak current on the square root of the scan rate v and their correlation coefficients for a1, a1′, a2, and c1 peaks for H_2_**L**, according to Figure 4b; Table S7: Equations for the dependences of peak potential and peak current on the scan rate v and their correlation coefficients for a1, a1′, and c1 peaks, according to Figure 8a; Table S8: Equations for the dependences of peak current on the square root of the scan rate v and their correlation coefficients for a1, a1′, and c1 peaks, according to Figure 8b; Table S9: Equations for the dependences of peak potential on the logarithm of the scan rate in CVs performed at different anodic limits; Table S10: Equations for the dependences of peak current on the square root of the scan rate in CVs performed at different anodic limits.
