# Supplementary material for: Ni(II)-Salophen—Comprehensive Analysis on Electrochemical and Spectral Characterization and Biological Studies
Source: Molecules. 2023 Jul 17;28(14):5464. doi: 10.3390/molecules28145464 (PMC10384438; doi:10.3390/molecules28145464)
Supplement: Supplementary file 1 [file molecules-28-05464-s001.zip › molecules-2470566-supplementary.pdf]

Supplementary Materials

Ni(II)-salophen - new aspects on electrochemical and spectral characterization and biological studies

Francis Aurelien Ngounoue Kamga, Madalina-Marina Hrubaru, Oana Enache, Elena Diacu, Constantin Draghici, Victorita Tecuceanu, Eleonora-Mihaela Ungureanu, Stephanie Nkemone and Peter Teke Ndifon

Table S1. Potential (E) and current (i) of the anodic (a1-a2), cathodic (c1-c2), and their related peaks in the reverse scans from CV and DPV, and half-wave potential ($E_{1/2}$) from RDE experiments for H_2**L** (1 mM in 0.1 M TBAP/CH_3CN).

Peak\ Parameter	E_{CV}(V)	i_{CV} (A)	E_{DPV}(V)	i_{DPV} (A)	$E_{1/2}$ (V)
a1	0.820	2.56×10^{-5}	0.766	5.87×10^{-6}	-
a2	1.623	9.51×10^{-5}	1.517	4.13×10^{-6}	-
a1′	0.409	-4.65×10^{-6}	-	-	-
c1	-1.958	5.26×10^{-5}	-1.898	8.97×10^{-6}	-1.931
c2	-2.698	8.04×10^{-5}	-2.231	2.11×10^{-6}	-
c1′	-1.962	-2.71×10^{-6}	-	-	-
c2′	-2.417	-7.14×10^{-6}	-	-	-

Table S2. Influence of the scan rate on the potential (E) and current (i) of the anodic (a1-a2) and cathodic (c1- c2) peaks in CV scans for H_2**L** (1 mM in 0.1 M TBAP/ CH_3CN).

v(V.s^{-1})	E_{c1}(V)	i_{c1} (A)	E_{a1}(V)	i_{a1} (A)	$E_{a1'}$(V)	$i_{a1'}$(A)	E_{c2}(V)	i_{c2}(V)
0.05	-1.923	3.49×10^{-5}	0.436	7.18×10^{-6}	0.412	3.33×10^{-7}	0.752	2.29×10^{-5}
0.1	-1.941	4.87×10^{-5}	0.447	9.37×10^{-6}	0.409	-5.49×10^{-7}	0.760	3.09×10^{-5}
0.2	-1.954	6.46×10^{-5}	0.464	1.32×10^{-5}	0.405	-1.72×10^{-6}	0.774	4.32×10^{-5}
0.3	-1.962	7.78×10^{-5}	0.477	1.66×10^{-5}	0.400	-2.57×10^{-6}	0.779	5.25×10^{-5}
0.5	-1.967	1.08×10^{-4}	0.502	2.38×10^{-5}	0.391	-4.70×10^{-6}	0.798	7.25×10^{-5}

Table S3. Potential (E) and current (i) of the anodic (a1-a2), cathodic (c1-c2), and their related peaks in the reverse scans from CV and DPV, and half-wave potential ($E_{1/2}$) from RDE experiments for Ni(II)**L** complex (0.4 mM in 0.1 M TBAP/CH_3CN).

Peak\ Parameter	E_{CV}(V)	i_{CV}(A)	E_{DPV}(V)	i_{DPV}(A)	$E_{1/2}$ (V)
a1	0.714	$3.351\ 1 \times 0^{-5}$	0.609	1.095×10^{-5}	-
a2	-	-	0.921	5.577×10^{-5}	-
a1′	-0.420	2.034×10^{-5}	-	-	-
a3	-	-	1.163	4.129×10^{-6}	-
a4	1.525	6.244×10^{-5}	1.432	4.903×10^{-6}	-
a5	1.773	9.230×10^{-5}	1.639	6.25×10^{-6}	-
c1	-1.817	1.728×10^{-5}	-1.788	4.171×10^{-6}	-1.784
c2	-2.466	1.907×10^{-5}	-2.446	7.298×10^{-6}	-
c1′	-1.750	3.570×10^{-5}	-	-	-
c2′	-2.354	-5.51×10^{-5}	-	-	-

Table S4. Influence of the scan rate on the potential (E) and current (i) of a1 and a1′peaks in anodic scans and c1 peak in cathodic scans in CV experiments for Ni(II)**L** complex (0.4 mM in 0.1 M TBAP/CH_3CN).

v(V.s^{-1})	E_{c1}(V)	$-i_{c1}$ (A)	E_{a1}(V)	i_{a1} (A)	$E_{a1'}$(V)	$i_{a1'}$(A)
0.05	-1.810	1.25×10^{-5}	0.680	4.67×10^{-5}	0.368	-1.21×10^{-5}
0.1	-1.820	1.91×10^{-5}	0.705	6.52×10^{-5}	0.371	-2.25×10^{-5}
0.2	-1.823	2.45×10^{-5}	0.727	8.95×10^{-5}	0.367	-2.91×10^{-5}
0.3	-1.834	3.01×10^{-5}	0.755	1.11×10^{-4}	0.366	-4.37×10^{-5}
0.5	-1.837	4.04×10^{-5}	0.798	1.43×10^{-4}	0.347	-6.64×10^{-5}

Table S5. Equations for the dependences of peak potential (Ep) and peak current (ip) on the scan rate v (in V/s) and their correlation coefficients for a1, a1′, a2, and c1 peaks for H_2**L**, according to Figure 4a.

Peak	Equation for Ep (V) vs log v	Correlation coefficient
a1	Epa1 (V) = 0.507 + 0.062· log v	0.974
a1′	Epa1′ (V) = 0.378 -0.022· log v	-0.946
a2	Epa2 (V) = 0.820 + 0.029· log v	0.964
c1	Epc1 (V) = -1.977 -0.044· log v	-0.985

Table S6. Equations for the dependences of peak current (ip) on the square root of the scan rate v (in V/s) and their correlation coefficients for a1, a1′, a2, and c1 peaks for H_2**L**, according to Figure 4b.

Peak	Equation for ip(A) vs $v^{1/2}$	Correlation coefficient
a1	ipa1(A) = $-1.252 \cdot 10^{-6} + 3.412 \cdot 10^{-5} \cdot v^{1/2}$	0.992
a1′	ipa1′(A) = $2.120 \cdot 10^{-6} -9.143 \cdot 10^{-6} \cdot v^{1/2}$	-0.980
a2	ipa2(A) = $-1.041 \cdot 10^{-6} + 1.014 \cdot 10^{-4} \cdot v^{1/2}$	0.997
c1	ipc1(A) = $-6.866 \cdot 10^{-7} -1.477 \cdot 10^{-4} \cdot v^{1/2}$	-0.995

Table S7. Equations for the dependences of peak potential (Ep) and peak current (ip) on the scan rate v (in V/s) and their correlation coefficients for a1, a1′ and c1 peaks, according to Figure 8a ([Ni(II)**L**] = 0.4 mM).

Peak	Equation Ep (V) vs log v(V/s)/ ip(A) vs $v^{1/2}$	Correlation coefficient
a1	Epa1 (V) = 0.819 + 0.110· log v	0.976
a1′	Epa1′ (V) = -0.911 + 0.773· log v	-0.911
c1	Epc1 (V) = -1.827 -0.016· log v	-0.970

Table S8. Equations for the dependences of peak current (ip) on the square root of the scan rate v (in V/s) and their correlation coefficients for a1, a1′ and c1 peaks, according to Figure 8b ([Ni(II)**L**] = 0.4 mM).

Peak	Equation (ip(A) = ai + bi·$v^{1/2}$; v in V/s)	Correlation coefficient
a1	ipa1(A) = $1.995 \cdot 10^{-6} + 1.988 \cdot 10^{-4} \cdot v^{1/2}$	0.999
a1′	ipa1′(A) = $1.409 \cdot 10^{-5} -1.091 \cdot 10^{-4} \cdot v^{1/2}$	-0.985
c1	ipc1(A) = $-3.237 \cdot 10^{-7} -5.576 \cdot 10^{-5} \cdot v^{1/2}$	-0.997

Table S9. Equations for the dependences of a1 and a1′ peaks potentials on the logarithm of the scan rate in CVs performed at different anodic limits.

Anodic limit scan (V)	Peak	Equation for Ep (V) vs log v	Correlation coefficient
0.7	a1	-	
0.9	a1	Epa1 (V) = 0.821 + 0.074· log v	0.981
1.12	a1	Epa1 (V) = 0.820 + 0.113· log v	0.972
0.7	a1′	Epa1′ (V) = 0.366 +0.007· log v	0.995
0.9	a1′	Epa1′ (V) = 0.338 -0.024· log v	-0.937
1.12	a1′	Epa1′ (V) = 0.343 -0.023· log v	-0.784

Table S10. Equations for the dependences of peak current on the square root of the scan rate (in V/s) in CVs performed at different anodic limits ([Ni(II)**L**] = 0.4 mM).

Anodic limit scan (V)	Peak	Equation for ip(A) vs $v^{1/2}$	Correlation coefficient
0.7	a1	--	
0.9	a1	$ipa1(A) = 2.401 \cdot 10^{-6} + 2.171 \cdot 10^{-4} \cdot v^{1/2}$	0.999
1.12	a1	$ipa1(A) = 1.792 \cdot 10^{-6} + 1.997 \cdot 10^{-4} \cdot v^{1/2}$	0.999
0.7	a1′	$ipa1'(A) = -4.897 \cdot 10^{-8} -8.079 \cdot 10^{-6} \cdot v^{1/2}$	-0.992
0.9	a1′	$ipa1'(A) = 6.113 \cdot 10^{-6} -6.637 \cdot 10^{-5} \cdot v^{1/2}$	-0.993
1.12	a1′	$ipa1'(A) = 1.404 \cdot 10^{-5} -1.095 \cdot 10^{-4} \cdot v^{1/2}$	-0.985

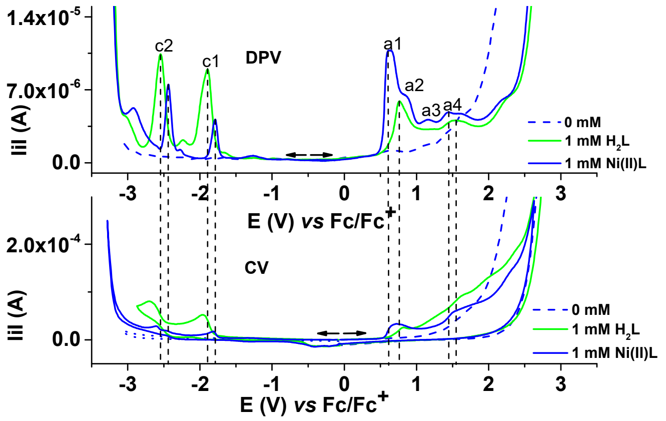

Figure S1. Anodic and cathodic DPV (up) and CV 0.1 V/s (down) curves on GC of H_2**L** and Ni(II)**L** complex superimposed at 1 mM concentrations in 0.1 M TBAP/CH_3CN; the cathodic currents are shown in absolute values.

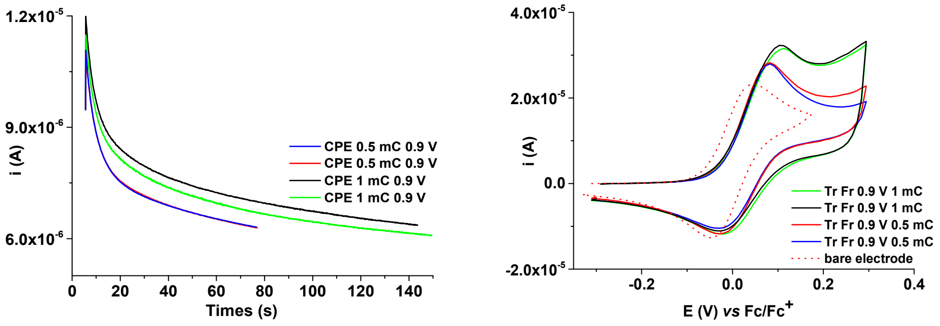

(a) **(b)**

Figure S2. (**a**) Chronoamperograms during the preparation of Ni(II)**L**-CMEs in 0.4 mM solution of Ni(II)**L** in 0.1 M TBAP/CH_3CN, by CPE at different charges; (**b**) CV curves (0.1 V/s) after their transfer in ferrocene solution (1 mM) in 0.1 M TBAP/CH_3CN vs. bare electrode.

(a)
0.7 V

(b)
0.9 V

(c)
1.13 V

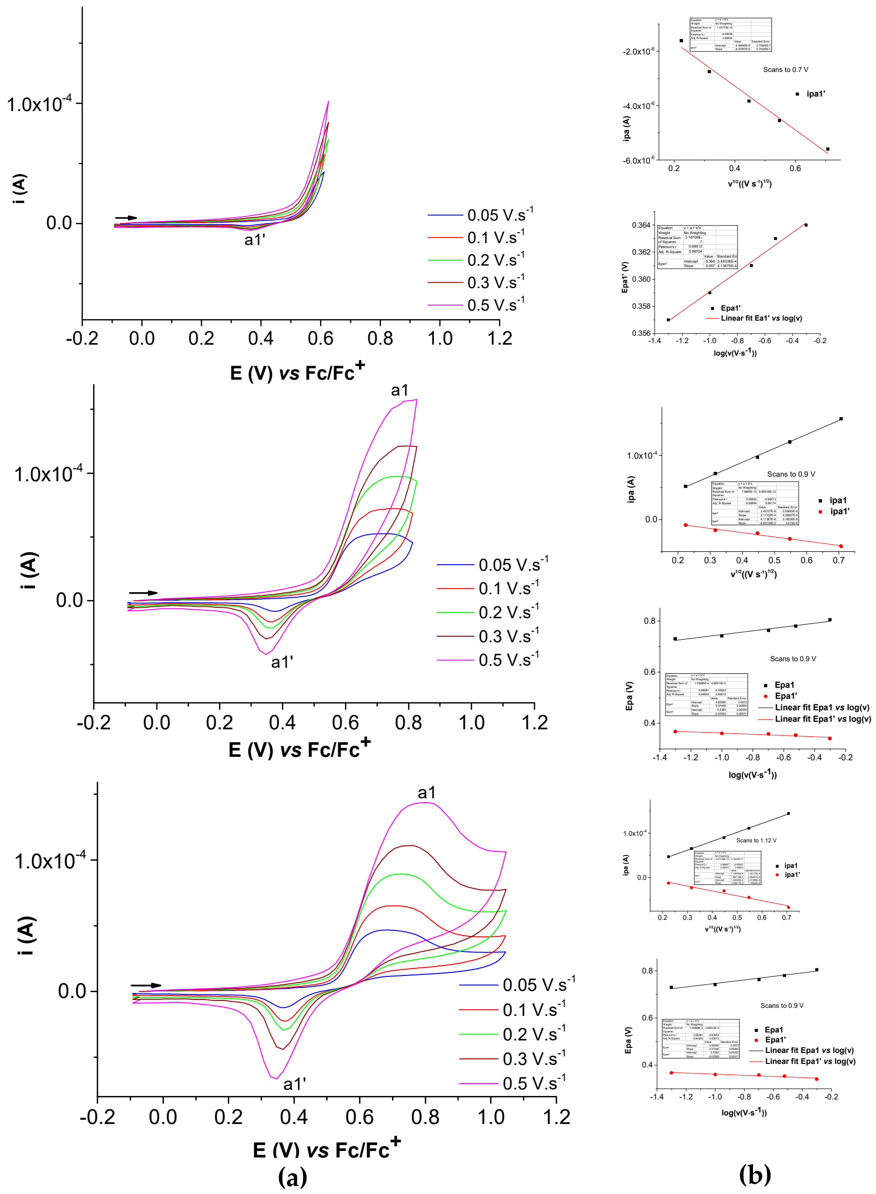

Figure S3. (**a**) Anodic cyclic voltammograms with anodic scan limits (*vs*. RE) of 0.7 V (a), 0.9 V (b), 1.13 V (c) at different scan rates for solution of [Ni(II)**L**] = 0.4 mM in 0.1 M TBAP/CH_3CN; (**b**) linear dependences of a1 and a1′ peaks currents on the square root of the scan rate, and linear dependences of a1 and a1′ peaks potentials on the logarithm of the scan rate.
